# Simultaneous double primary malignant tumors of MSS/pMMR ascending colon cancer and MSI-H/dMMR duodenal cancer with nearly 2 years of recurrence-free survival after MDT-guided comprehensive treatment: a case report

**DOI:** 10.3389/fonc.2025.1573580

**Published:** 2025-05-30

**Authors:** Weimin Wang, Quan Yang, Yifan He, Xuefeng Cha, Chaoxian Xiong, Xiaoxia Li, Jie Li, Pin Gao, Kun Yu

**Affiliations:** Department of Colorectal Surgery, Yunnan Cancer Hospital, The Third Affiliated Hospital of Kunming Medical University, Kunming, China

**Keywords:** synchronous double primary cancers, colorectal cancer, small intestinal cancer, multidisciplinary team (MDT), case report

## Abstract

The exceedingly rare clinical presentation of synchronous ascending colon carcinoma with duodenal adenocarcinoma demonstrating discordant mismatch repair protein expression patterns forms the cornerstone of this investigation. Through detailed analysis of a unique case featuring co-occurring MSS/pMMR ascending colon adenocarcinoma and MSI-H/dMMR duodenal adenocarcinoma, this study demonstrates the successful implementation of multidisciplinary therapeutic protocols achieving 22-month progression-free survival post-radical resection. These clinical findings offer empirical evidence for optimizing clinical management while systematically addressing diagnostic complexities and treatment dilemmas inherent to synchronous dual primary intestinal carcinomas. Particular emphasis is placed on reconciling therapeutic conflicts arising from differential tumor biology and developing precision strategies for molecularly heterogeneous synchronous malignancies.

## Introduction

Colorectal malignancies, constituting a formidable public health challenge on a global scale, have persistently ranked among the leading causes of cancer-related morbidity and mortality. This alarmingly high prevalence stands in stark contrast to the clinical rarity of small intestinal neoplasms, which account for merely 1-2% of gastrointestinal malignancies. The co-occurrence of colorectal and small intestinal dual primary carcinomas represents an exceptionally uncommon clinical phenomenon, with no documented cases exhibiting discordant mismatch repair (MMR) protein expression patterns across anatomically distinct tumor sites. This knowledge gap has hindered the establishment of standardized therapeutic protocols for such complex clinical presentations. Notably, emerging immune-modulatory strategies demonstrate promising yet unvalidated therapeutic potential in managing synchronous primary intestinal carcinomas.

**Figure 1:**
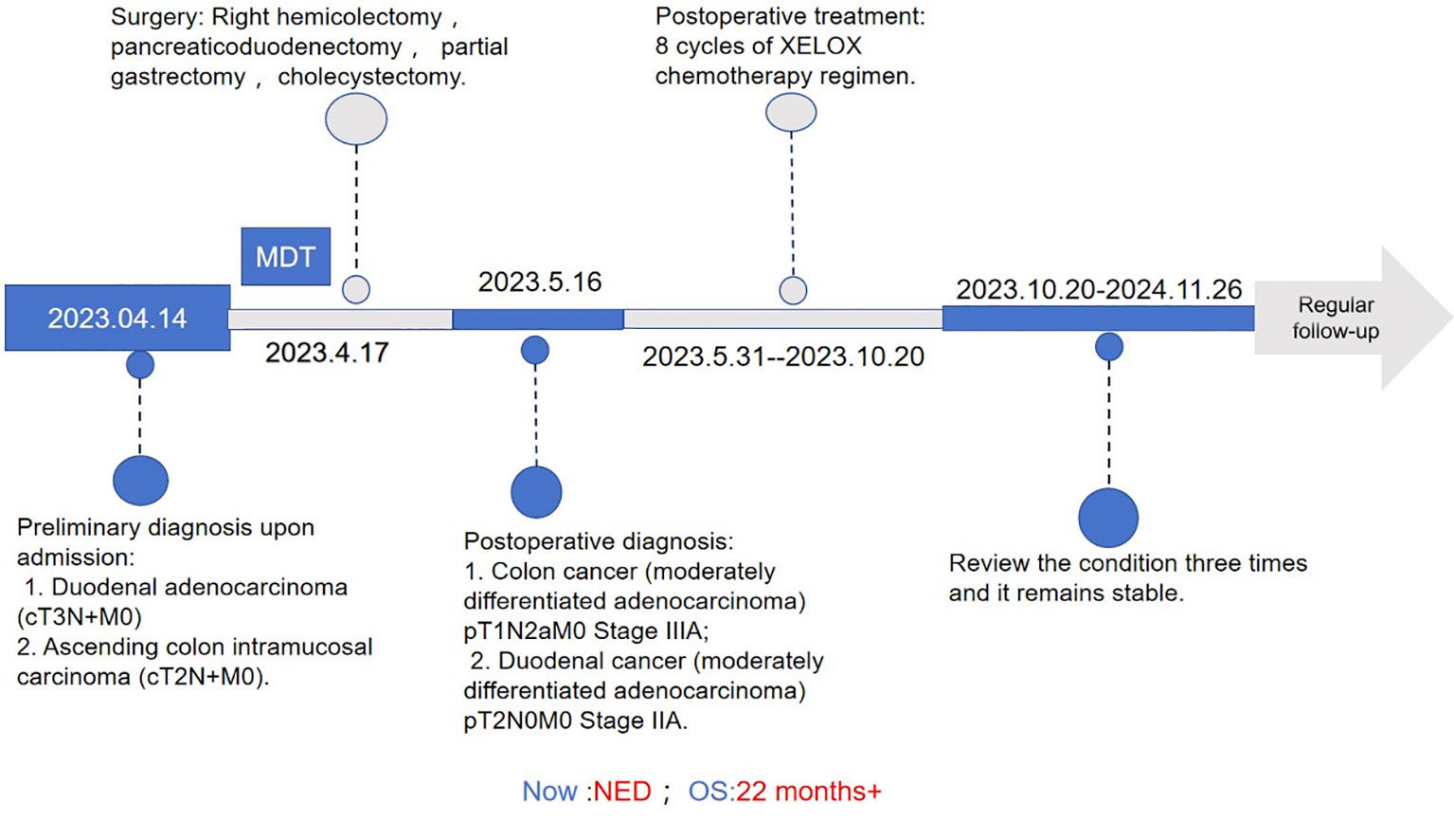
Treatment process flow chart for this case. MDT, multidisciplinary team; XELOX,Capecitabine (Xeloda) + Oxaliplatin combination chemotherapy regimen.NED, no evidence of disease; OS, overall survival.

This investigation pioneers the documentation of a 73-year-old female presenting with molecularly distinct synchronous malignancies: a microsatellite-stable/pMMR ascending colon adenocarcinoma coexisting with a MSI-H/dMMR duodenal adenocarcinoma. Following radical surgical intervention guided by multidisciplinary tumor board recommendations, the patient maintained disease-free status throughout 22 months of postoperative surveillance(**Figure 1**). The clinical significance of this case manifests in two key dimensions: 1) The therapeutic quandary posed by divergent molecular profiles in synchronous primaries, and 2) The paradigm-shifting implications of differential treatment responses between immunologically “hot” (MSI-H/dMMR) and “cold” (MSS/pMMR) tumors to checkpoint inhibitors versus conventional chemotherapy. This study methodically examines critical clinical considerations spanning hereditary cancer syndrome screenings, temporal optimization of targeted therapies, and longitudinal biomarker monitoring. Particular emphasis is placed on molecularly-guided precision immunotherapy, encompassing PD-1 inhibitor application in MSI-H/dMMR neoplasms and epigenetic modulation strategies to overcome immune desertification in MSS/pMMR tumors. These insights provide new theoretical foundations and practical paradigms for managing highly heterogeneous dual primary malignancies.

## Case introduction

**Figure 2:**
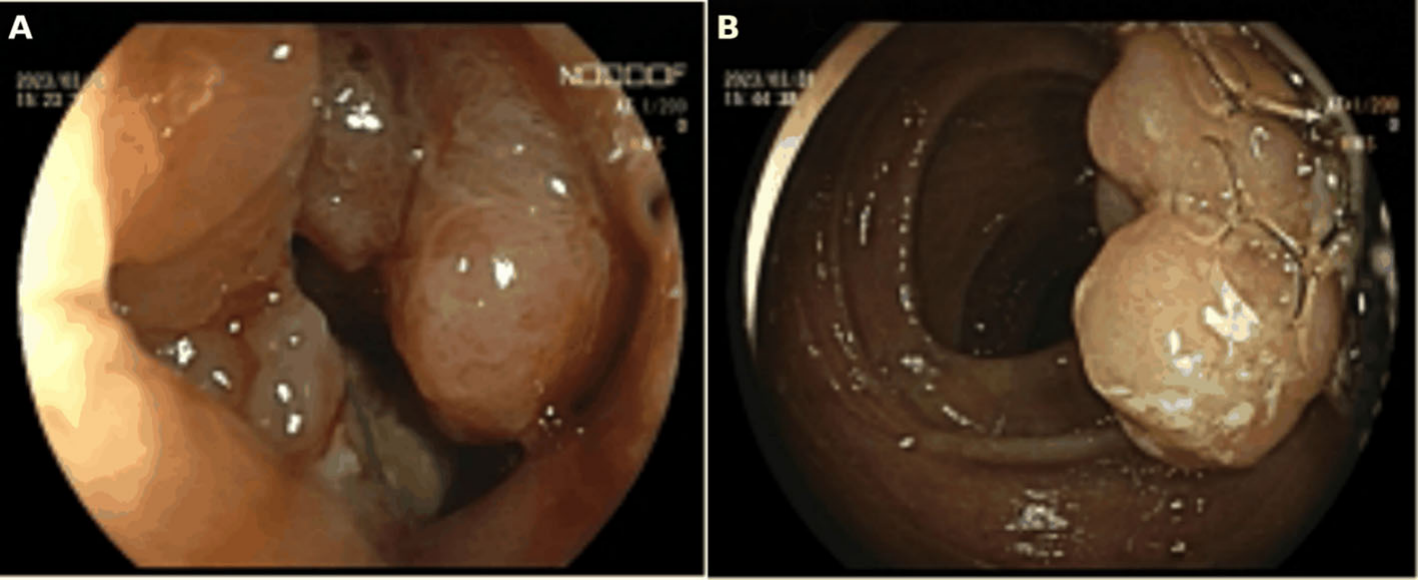
**(A)** Preoperative gastrointestinal endoscopy indicated a tumor in the duodenal bulb.**(B)** Preoperative gastrointestinal endoscopy indicated a tumor at the hepatic flexure of the ascending colon.

**Figure 3:**
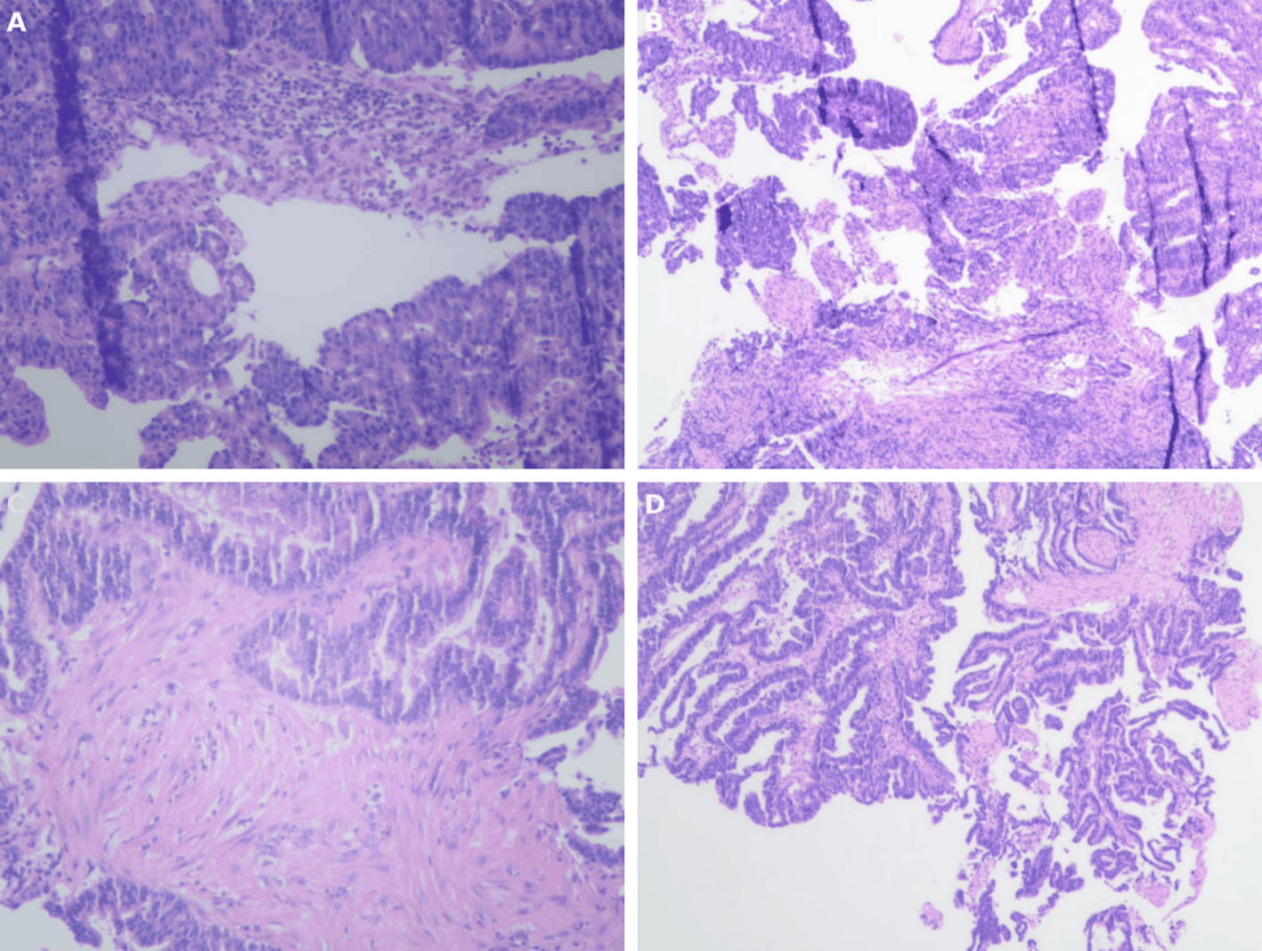
**(A,B)** The preoperative gastrointestinal endoscopic biopsy HE staining results of the tumor in the ascending colon indicate intramucosal adenocarcinoma of the ascending colon.**(C,D)** Preoperative gastrointestinal endoscopic biopsy HE staining results of the tumor in the duodenal bulb indicate adenocarcinoma of the duodenal bulb.

**Figure 4:**
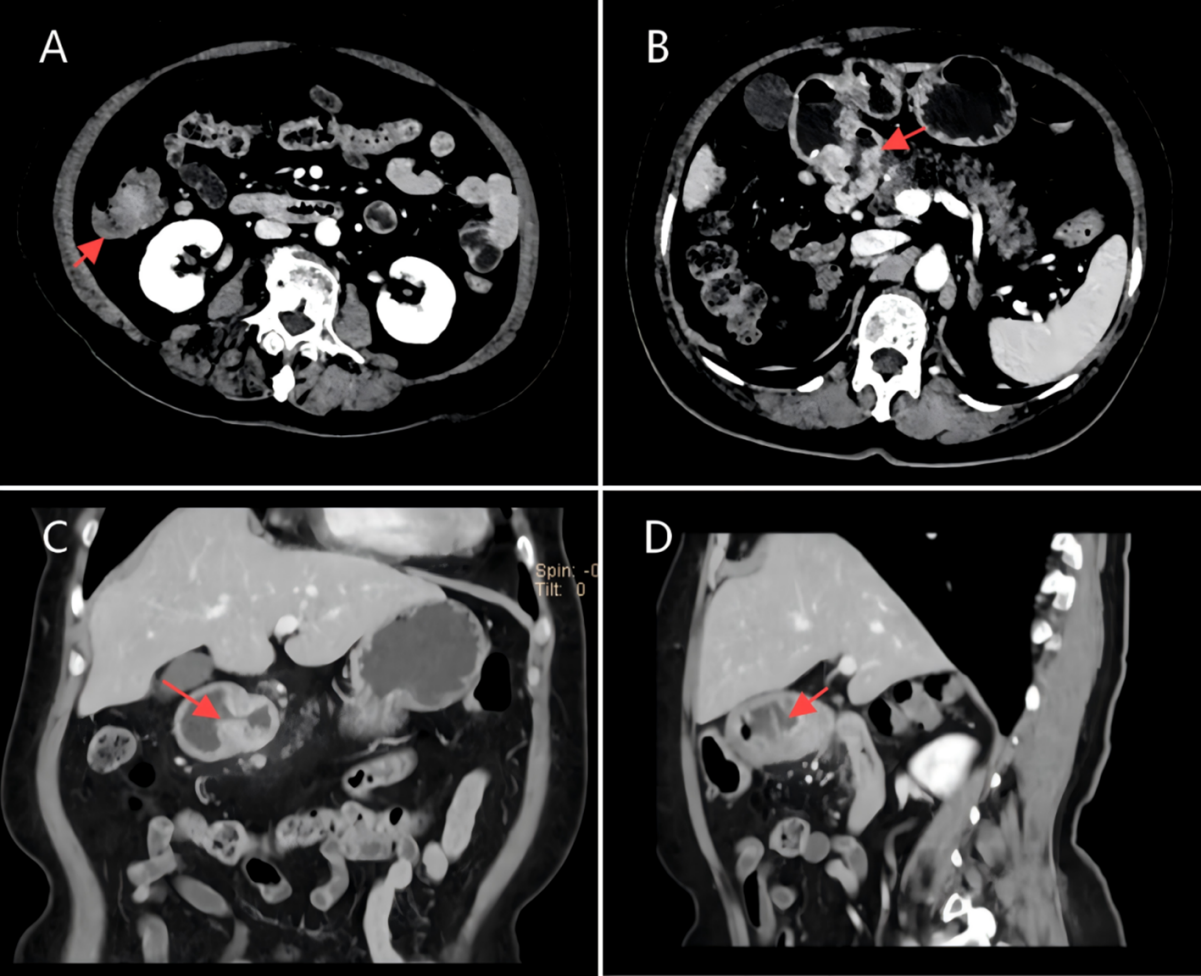
**(A,D)** CT scan image of a tumor in the ascending colon, dated April 7, 2023. **(B,C)** CT scan image of a tumor in the duodenal bulb, dated April 7, 2023.

The patient is a 73-year-old female who was admitted on April 14, 2023, due to “intermittent abdominal pain for more than 1 year, worsening for over a month.” Prior to admission, a gastrointestinal endoscopy at a local hospital revealed: “1. Duodenal bulb ulcer (active phase), nature pending, with bleeding and deformation of the bulb; 2. Multiple polyps in the gastric fundus and body; 3. Lesion in the hepatic flexure of the colon, nature pending; 4. Internal hemorrhoids.” Biopsy results showed: “1. (Duodenal bulb) ulcer with high-grade intraepithelial neoplasia; 2. (Hepatic flexure) tubular villous adenoma with low-grade intraepithelial neoplasia.” The patient was referred to Yunnan Cancer Hospital for further treatment. Past medical history: In 2018, the patient underwent cardiac radiofrequency ablation at Yunnan Provincial Cardiovascular Disease Hospital. Upon admission, physical examination revealed a soft abdomen with no scars or masses, no tenderness or rebound tenderness in the abdomen, and no abnormal abdominal masses palpated. Digital rectal examination (in the chest-knee position) revealed no abnormal masses in the rectal cavity up to approximately 6 cm. The rectal mucosa appeared smooth, and there was no blood on the glove after withdrawal. A follow-up gastrointestinal endoscopy at Yunnan Cancer Hospital showed: A nodular mass with deep ulceration, narrow and stiff at the duodenal bulb (**Figure 2A**), with biopsy pathology confirming duodenal bulb adenocarcinoma (**Figures 3C, D**). Immunohistochemical results were: MLH1 (+), MSH2 (+), MSH6 (+), PMS2 (tumor cells -, stromal cells +). At the hepatic flexure of the colon (**Figure 2B**), a 3.0x3.0 cm lobulated adenoma-like polyp was seen with ulceration, hard texture, and poor elasticity, with biopsy pathology confirming mucosal carcinoma of the ascending colon (**Figures 3A, B**). Immunohistochemical results were: MLH1 (+), MSH2 (+), MSH6 (+), PMS2 (+).Abdominal enhanced CT (**Figure 4**) showed: uneven thickening of the duodenal wall, suspicious for malignancy, with multiple lymph nodes around the intestines, possibly indicating metastasis; a nodule in the ascending colon. Abdominal MRI revealed: uneven thickening of the duodenal bulb and descending segment wall with multiple lymph nodes around the intestines, indicative of malignancy; a nodule in the ascending colon. Carcinoembryonic antigen (CEA) was 9.46 ng/mL. Clinical diagnosis:1. Duodenal adenocarcinoma (cT3N+M0);2. Mucosal carcinoma of the ascending colon (cT2N+M0);3.Dual primary cancers of the duodenum and ascending colon (synchronous).

A multidisciplinary team (MDT) discussion was conducted at Yunnan Cancer Hospital, involving specialists from colorectal surgery, radiology, pathology, medical oncology, and hepatobiliary-pancreatic surgery departments. The main opinions were as follows: Based on the patient’s imaging and pathological data, the patient was considered to have synchronous double primary cancers of the duodenum and ascending colon. The immunohistochemistry results of the patient’s duodenum and right hemicolon indicated different expressions of mismatch repair proteins between the two sites. During the treatment strategy formulation process, the team assessed the patient’s specific condition: duodenal adenocarcinoma (cT3N+M0), mucosal carcinoma of the ascending colon (cT2N+M0), ECOG score of 2, and a history of cardiac radiofrequency ablation 5 years prior. The team used the American Society of Anesthesiologists (ASA) classification to evaluate the patient’s surgical risk as grade III. Comparative analysis of treatment options showed: considering that the patient was an elderly female, simultaneous surgical resection would likely exceed 4 hours, increasing the risk of intraoperative bleeding and postoperative complications (such as anastomotic leakage and abdominal infection) to 15-20%; whereas staged surgery might delay the second operation due to prolonged recovery from the first operation (estimated 6–8 weeks), leading to tumor progression and missing the optimal surgical timing, and the cumulative risk of damage to cardiopulmonary function from two anesthesia procedures would be higher. The team developed a strict perioperative management plan, including albumin supplementation 3 days before surgery, bowel preparation the day before surgery (polyethylene glycol electrolyte powder + metronidazole), application of Enhanced Recovery After Surgery (ERAS) concepts during the operation, intraoperative warming measures, strict control of fluid infusion, and early enteral nutrition support after surgery. After multiple discussions by the expert group and thorough communication with the patient and family members, explaining the advantages and disadvantages of both approaches in detail, the final decision was made to proceed with simultaneous surgical resection.

**Figure 5:**
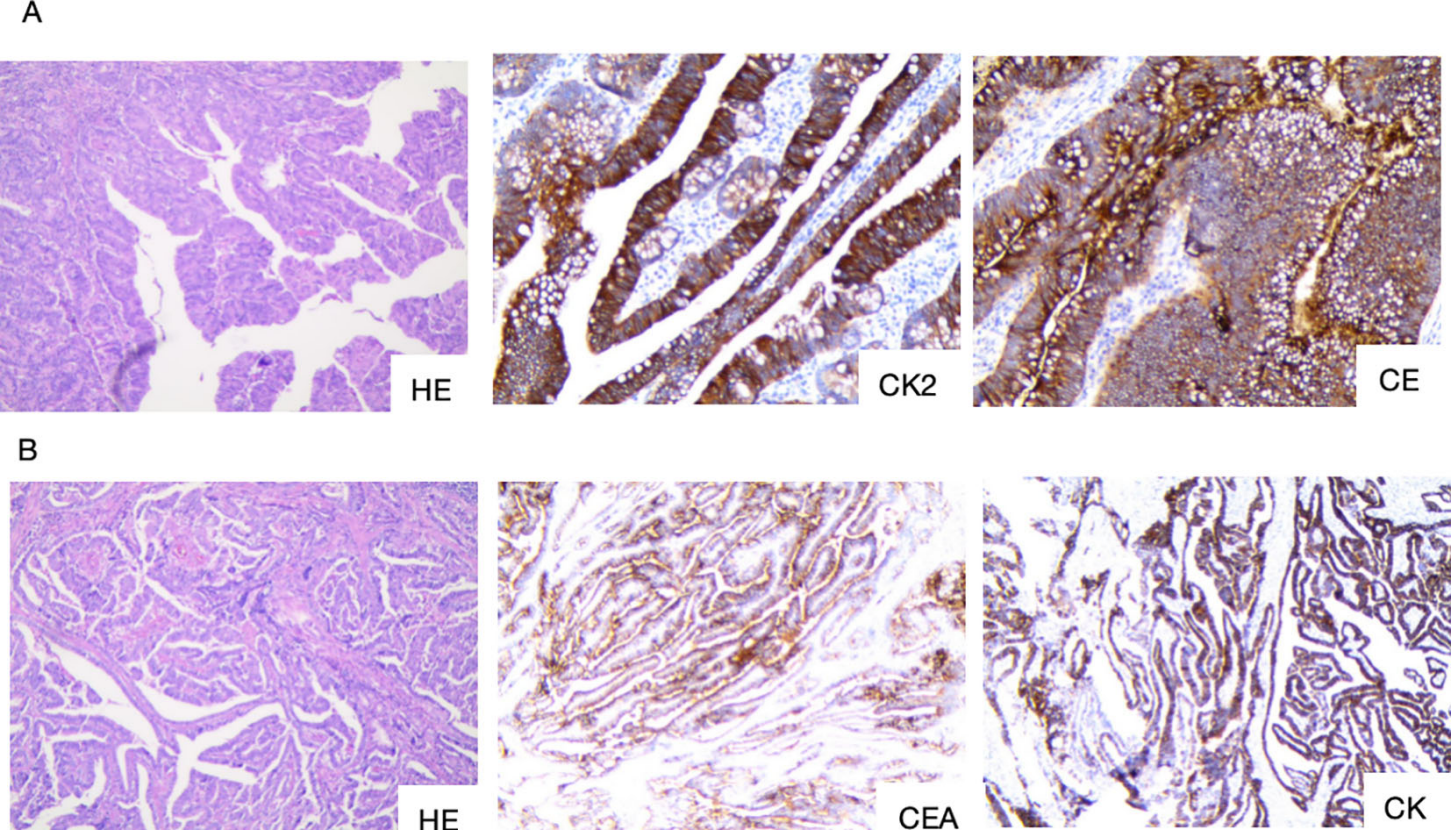
**(A)** Postoperative pathology of the right hemicolon and tumor site. **(B)** Postoperative pathology of the pancreatoduodenal region and duodenal tumor site. HE, staining of the tumor tissue; CK20, Cytokeratin 20; CEA, Carcinoembryonic antige; CK7, Cytokeratin 7.

After preoperative preparation and anesthesia evaluation, on April 17, 2023, the patient underwent right hemicolectomy, duodenectomy, and partial gastrectomy under general anesthesia. Intraoperative exploration showed that the duodenal tumor invaded and adhered closely to the head of the pancreas. After evaluation by hepatobiliary and pancreatic surgery specialists, the patient underwent right hemicolectomy, pancreaticoduodenectomy, and cholecystectomy. Postoperative pathology report (**Figure 5**):1. Right hemicolon and tumor: moderately differentiated adenocarcinoma, with cancer infiltrating the submucosa of the colon wall. No cancer tissue was observed at the resection margins of the colon, ileum, or bile duct. Mesenteric lymph nodes (6/13) showed cancer metastasis. Immunohistochemical results: CK7 (-), CK20 (+), CDX2 (+), Villin (+), CEA (+), PAX8 (-), P16 (partial +), P53 (+), WT-1 (-), Ki-67 (+, approximately 60%);2. Pancreaticoduodenal and duodenal tumors: moderately differentiated adenocarcinoma, with cancer tissue infiltrating the full thickness of the intestinal wall. No cancer tissue was observed in the pancreatic and gastric specimens. Lymph nodes around the stomach (0/4) and mesenteric lymph nodes (0/4) did not show cancer metastasis. Lymph nodes in groups “7, 8, 9” (0/3) and “16” (0/6) did not show cancer metastasis. Lymph nodes in group “12” showed fibrofatty tissue without lymph node structure or cancer tissue. Immunohistochemical results: CK7 (+), CK20 (partial +), CDX2 (-), Villin (+), CEA (+), PAX8 (-), P16 (+), P53 (-), WT-1 (-), Ki-67 (approximately 40%+), ER (-), PR (-).Postoperative genomic testing results: The duodenal tumor showed the following clinically significant mutations (malignant tumor cells in the tested sample: 60%, quality control standard ≥20%): PMS2 mutation (abundance: 26.83%), APC mutation (abundance: 40.78%), ARID1A mutation (abundance: 27.63%), ATM mutation (abundance: 29.98%), BRCA2 p.N1784fs mutation (abundance: 28.51%), BRCA2 p.N986fs mutation (abundance: 27.27%), ERBB2 mutation (abundance: 31.00%), NF1 mutation (abundance: 25.00%), tumor microsatellite stability: MSI-H (detection method: NGS); The ascending colon tumor showed the following clinically significant mutations (malignant tumor cells in the tested sample: 35%, quality control standard ≥20%): BRAF V600E mutation (abundance: 15.79%), APC mutation (abundance: 34.95%), SMAD4 mutation (abundance: 16.44%) and TP53 mutation (abundance: 16.41%), tumor microsatellite stability: MSS (detection method: NGS). Postoperative diagnosis and staging: (1) Moderately differentiated adenocarcinoma of the colon (pT1N2aM0 Stage IIIA); (2) Moderately differentiated adenocarcinoma of the duodenum (pT2N0M0 Stage IIA). Additionally, postoperative supplementary 2B3D method was performed to confirm the MSI status, which verified that the duodenal tumor microsatellite stability is MSI-H, while the ascending colon tumor microsatellite stability is MSS.

**Figure 6:**
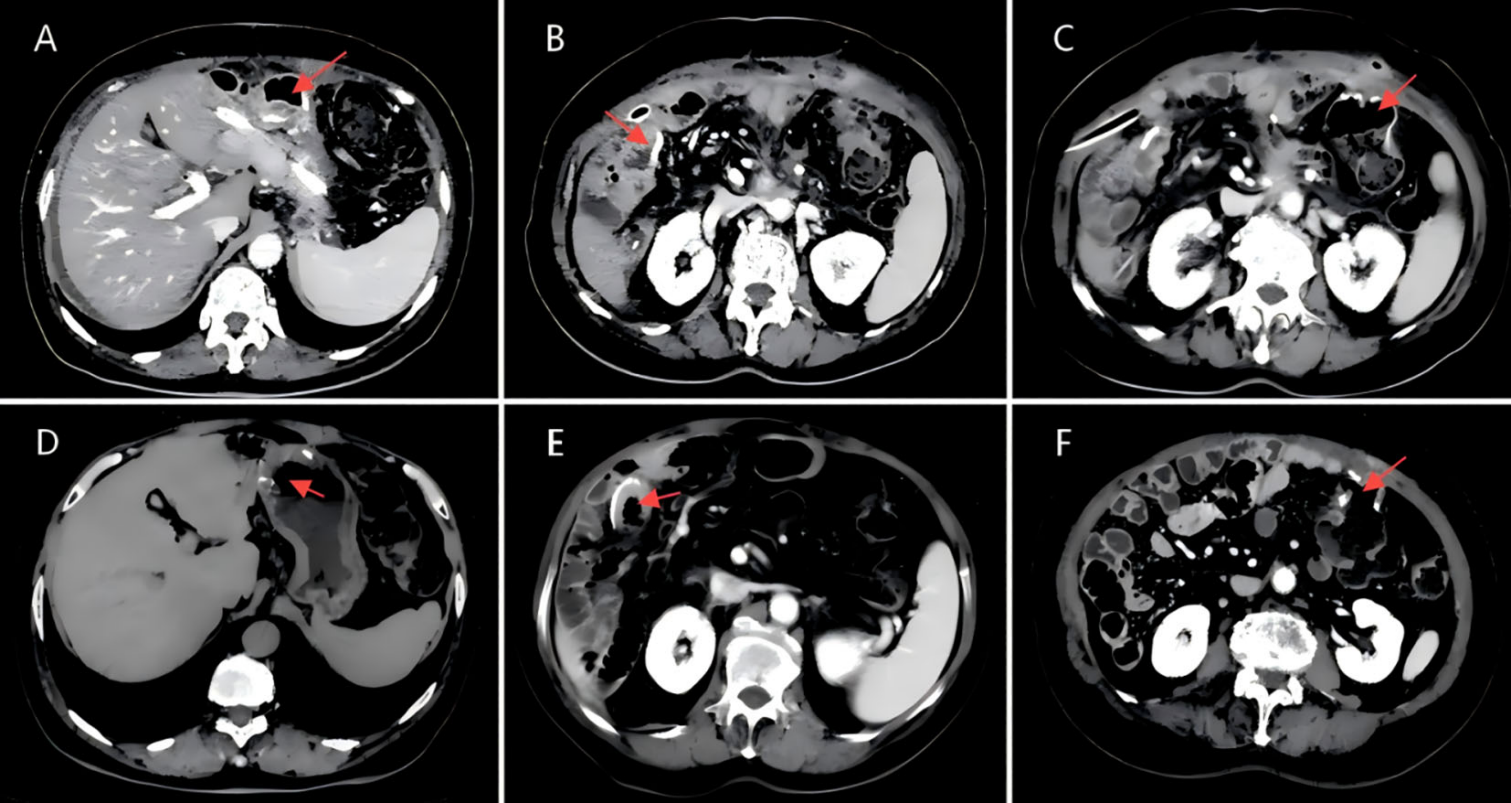
**(A)**CT scan image of the gastro-intestinal anastomosis, dated March 19, 2024.**(B)**CT scan image of the biliary-enteric anastomosis, dated March 19, 2024.**(C)**CT scan image of the ileocolic anastomosis (ileum to transverse colon), dated March 19, 2024.**(D)**CT scan image of the gastro-intestinal anastomosis, dated November 26, 2024.**(E)**CT scan image of the biliary-enteric anastomosis, dated November 26, 2024.**(F)**CT scan image of the ileocolic anastomosis (ileum to transverse colon), dated November 26, 2024.

After surgery, the patient gradually resumed eating following total parenteral nutrition and other symptomatic supportive treatments, and was discharged after suture removal. A multidisciplinary discussion was held at Yunnan Cancer Hospital after the operation, with the following specific opinions: The patient was confirmed by pathology and genomic testing to have double primary cancers of the duodenum and ascending colon. Although the patient’s duodenal tumor was confirmed to be MSI-H, the current application of immunotherapy in MSI-H tumors is mainly focused on advanced/metastatic disease or as neoadjuvant therapy before surgery, and has not yet received clear guideline recommendations for adjuvant treatment in early-stage tumors. In addition, considering that the patient also had MSS/pMMR ascending colon cancer, the MDT expert group conducted a comprehensive assessment, including the treatment needs of two tumors with different molecular characteristics, the patient’s overall condition, and chemotherapy tolerance. They unanimously agreed that the standard “XELOX” regimen was the most suitable adjuvant treatment option for this patient. This decision balanced treatment efficacy with toxic reactions and also considered the comprehensive benefits for both tumors. It was finally decided that the patient’s postoperative treatment plan would be 8 cycles of “XELOX” regimen systemic chemotherapy, with regular follow-up examinations. The first post-operative follow-up showed that CEA had returned to normal, and chest-abdomen-pelvis enhanced CT showed no lesions, suggesting a tumor-free state. The patient completed 8 cycles of “XELOX” regimen treatment from May 31, 2023, to October 12, 2023. Imaging follow-up until November 26, 2024, more than 1.5 years later, showed no signs of tumor recurrence or progression. The patient is currently still under regular outpatient follow-up examinations (**Figure 6**).

## Discussion

Primary colorectal cancer (CRC) combined with small bowel cancer (Small bowel adenocarcinoma, SBA) is rare in clinical practice. Epidemiological studies indicate that the age-standardized incidence rate of CRC worldwide is approximately 40–50 per 100,000, accounting for 9.8% of all malignant tumors (1, 2). In contrast, the incidence of small bowel cancer is much lower, with an age-standardized incidence rate of only 1–2 per 100,000, representing 3-6% of gastrointestinal cancers (3). Among these, duodenal adenocarcinoma accounts for approximately 40-50% of small bowel cancers (4). Notably, there have been no reported cases of synchronous primary CRC combined with duodenal adenocarcinoma exhibiting inconsistent mismatch repair (MMR) expression in global literature (5). Most reported cases of multiple primary gastrointestinal cancers are closely associated with hereditary syndromes, such as Lynch syndrome and Familial Adenomatous Polyposis (FAP) (6).

Regarding mismatch repair (MMR) expression, the proportion of deficient mismatch repair (dMMR) in CRC is approximately 15-20%, mainly seen in Lynch syndrome-associated CRC and some sporadic cases (7). In small bowel cancer, the dMMR rate is 20-30%, with duodenal adenocarcinoma showing a slightly lower dMMR rate compared to small bowel cancers in other locations, which is closely related to germline or somatic mutations in MMR genes (8). MMR status plays a significant role in the prognosis and treatment prediction for gastrointestinal cancers. dMMR tumors typically have a better prognosis and are more responsive to immune checkpoint inhibitor therapy, whereas proficient MMR (pMMR) tumors may require more aggressive chemotherapy regimens (9, 10). In CRC, dMMR status has become a crucial predictive indicator for immune therapy benefits, significantly improving patient prognosis.

The treatment strategy for colon cancer should consider disease staging, tumor location, overall patient condition, and molecular characteristics to develop a personalized treatment plan (11). For early-stage colon cancer (Stage I-II), surgical resection is the main treatment approach, with laparoscopic surgery becoming the standard procedure due to its lower postoperative complication rates. For T1N0M0 early lesions, endoscopic resection may be sufficient (12). For Stage II high-risk patients and Stage III patients, postoperative adjuvant chemotherapy is necessary. The IDEA study shows that the FOLFOX or CAPOX regimens are currently the most commonly used adjuvant chemotherapy treatments with strong evidence from clinical trials (12). Treatment strategies for advanced or metastatic colon cancer are more complex and require personalized selection based on molecular biomarkers (13). Studies have confirmed that RAS wild-type patients can benefit from anti-EGFR drugs (such as cetuximab and panitumumab), and anti-angiogenesis drugs like bevacizumab have also shown significant efficacy in specific patients (14).

In recent years, as immunotherapy has demonstrated significant potential in the treatment of advanced colon cancer, especially for MSI-H/dMMR patients, it has become an essential part of the systemic treatment for this subset of patients. The KEYNOTE-177 study showed that pembrolizumab as first-line treatment for MSI-H/dMMR metastatic colon cancer significantly prolonged progression-free survival (PFS) compared to chemotherapy, with a median PFS of 16.5 months in the pembrolizumab group, compared to only 8.2 months in the chemotherapy group (HR=0.60, P<0.001), and it also had a higher objective response rate (ORR of 43.8% vs. 33.1%) (15). The CheckMate-142 study further confirmed the benefits of immunotherapy, showing that nivolumab combined with ipilimumab demonstrated significant efficacy in MSI-H/dMMR colon cancer patients, with an ORR of 55% and a 1-year overall survival (OS) rate of 85% (16). Additionally, immunotherapy offers better tolerability and fewer adverse reactions compared to traditional chemotherapy, which lays an important foundation for its widespread application in colon cancer treatment (17).

Small bowel cancer, due to its unique anatomical location and relatively low incidence, presents a more complex treatment strategy (18). Compared to other gastrointestinal tumors, small bowel cancer is usually diagnosed at a later stage, which significantly affects treatment outcomes and prognosis (19). Currently, treatment plans are primarily based on tumor staging, histological type, and the patient’s individual condition. Surgical treatment is the main approach for localized small bowel adenocarcinoma. For resectable tumors, radical resection of the tumor and its corresponding lymphatic drainage areas is required. Duodenal cancer typically requires pancreaticoduodenectomy (Whipple procedure), while jejunal and ileal cancers require removal of the corresponding bowel segments (20). Adjuvant chemotherapy may benefit some high-risk patients, with the FOLFOX regimen still being the most commonly used option (21). For advanced or metastatic cases, systemic chemotherapy (such as FOLFOX, FOLFIRI, or CAPOX) remains the primary treatment modality (22). In the field of immunotherapy for small bowel cancer, treatment responses in MSI-H/dMMR subtype patients are particularly notable. Both the CheckMate-142 and KEYNOTE-158 studies have shown significant efficacy of PD-1 inhibitors (such as pembrolizumab and nivolumab) in these patients. The KEYNOTE-158 study results demonstrated that pembrolizumab had an ORR of 42% and a disease control rate (DCR) of 73% in MSI-H small bowel cancer patients, with durable efficacy, and some patients experienced remission for over two years (23). Furthermore, immunotherapy has shown good tolerability in small bowel cancer patients, further highlighting its potential for application in advanced cases (24).

In cases of dual primary cancers with colorectal cancer (CRC) and small bowel cancer, clinical decision-making presents significant challenges due to differences in the pathogenesis and physiological pathological characteristics of the two cancers. Treatment strategies must take into account the staging of both tumors, the patient’s overall condition, organ function reserve, and the expected survival benefit (25, 26). Research indicates that the prognosis for patients with dual primary cancers is poorer compared to those with a single primary cancer, with a significant reduction in the 5-year overall survival rate (27). In patients with overall acceptable conditions, simultaneous surgical resection is considered the preferred option. Multicenter retrospective studies have shown that, compared to staged surgery, simultaneous surgery does not significantly increase postoperative complication rates or perioperative mortality. It also effectively shortens overall hospital stay and treatment duration (28, 29). However, for elderly patients or those with severe underlying conditions, staged surgery remains a safer approach, with priority given to treating life-threatening lesions (30). In terms of systemic therapy, the choice of chemotherapy regimen should balance the sensitivity of the two tumors and the patient’s tolerance. A study involving patients with double primary gastrointestinal cancers showed that platinum-based chemotherapy regimens, such as FOLFOX or CAPOX, can effectively cover colon cancer and small bowel adenocarcinoma. According to several studies, platinum-based chemotherapy regimens have a good response for the treatment of these tumors, with an objective response rate (ORR) of 42.3% and a median progression-free survival (PFS) of 8.6 months (31, 32). For MSI-H/dMMR patients, immune checkpoint inhibitors show significant efficacy. The KEYNOTE-158 study revealed that pembrolizumab had an ORR of 42% in MSI-H small bowel cancer and demonstrated durable efficacy (33).

In this case, the mismatch repair status of the two tumors was different, which made the treatment of dual primary cancers more challenging, particularly when considering preoperative immunotherapy. Immune checkpoint inhibitors, including PD-1 inhibitors, have proven to be highly effective in MSI-H/dMMR tumors. However, their effectiveness in microsatellite stable (MSS) or proficient mismatch repair (pMMR) tumors is still uncertain (34). While preoperative immunotherapy may show a high response rate in MSI-H/dMMR tumors, it may not have a significant impact on MSS lesions and could potentially lead to tumor progression (35). Additionally, immunotherapy carries the risk of immune-related adverse events, such as colitis or hepatitis, which can increase surgical risks (36). As a result, the decision to use preoperative immunotherapy must carefully balance the potential benefits against the associated risks.

Preoperative neoadjuvant chemotherapy plays a significant clinical role in the treatment of multiple primary tumors, especially in cases of colon cancer combined with small bowel cancer. For tumors with microsatellite instability (MSI-H) or mismatch repair deficiency (dMMR), chemotherapy combined with immunotherapy has been shown to significantly reduce tumor burden and improve local tumor treatment responses. An important goal of neoadjuvant chemotherapy is to achieve complete clinical remission (cCR) by reducing the tumor volume as much as possible, which may even allow for the exemption of surgery or partial exemption from surgery. This treatment strategy not only significantly reduces the difficulty of surgery but also decreases the incidence of postoperative complications and lowers the physiological burden of surgery on the patient (9).

For patients with multiple primary tumors, especially in cases of colon cancer combined with small bowel cancer, preoperative neoadjuvant chemotherapy can simplify the surgical process to some extent. For example, neoadjuvant chemotherapy is first used to effectively shrink the colon tumor, minimizing its impact on the patient’s overall health, and then surgery for the colon tumor can be prioritized. Following this, based on treatment outcomes and molecular testing results, an appropriate immunotherapy regimen can be selected for subsequent treatment of the small bowel tumor. If the tumor has significantly shrunk, even reaching pathological complete remission (pCR), consideration can be given to continuing immunotherapy and delaying more invasive surgery, further reducing the patient’s treatment burden and postoperative recovery time (12).

However, it is worth noting that for pMMR/MSS-type tumors, the efficacy of immunotherapy remains unclear, particularly during the neoadjuvant treatment phase, where it is crucial to consider whether this type of tumor could progress. Existing studies suggest that pMMR/MSS tumors usually have a poor response to immunotherapy and may even develop resistance or progression due to immunotherapy (35). Therefore, when using immunotherapy in combination with neoadjuvant chemotherapy, it is essential to strictly evaluate the response of this type of tumor and consider the potential risk of tumor progression.

Regarding surgical strategy, if the patient’s overall condition permits, the primary lesion and resectable metastatic lesions should be removed first, particularly in cases with limited metastasis (37). If the surgery is difficult or simultaneous complete resection is not possible, staged surgery should be considered, prioritizing life-threatening lesions, followed by postoperative adjuvant therapy. In this process, imaging evaluation and dynamic monitoring of molecular biomarkers (such as CEA, CA19-9) can provide important guidance for subsequent treatment decisions (38).

Another point worth discussing in this case is that the patient’s preoperative gastrointestinal biopsy and immunohistochemistry showed negative PMS2 expression in tumor cells and positive expression in stromal cells. PMS2 (Postmeiotic Segregation Increased 2) is a crucial component of the DNA mismatch repair (MMR) system, and its loss of expression is often indicative of microsatellite instability (MSI), which plays a significant role in tumor development and treatment response. The clinical implications of PMS2 expression are considerable. When PMS2 is present in both tumor and stromal cells, it suggests that the MMR system remains intact, and the tumor is likely microsatellite stable (MSS). In such cases, patients tend to respond better to conventional chemotherapy but may have limited benefit from immune checkpoint inhibitors (39). On the other hand, if PMS2 expression is absent in both tumor and stromal cells, it typically indicates a more extensive MMR deficiency, suggesting an MSI-H status. Tumors with MSI-H generally exhibit a high tumor mutational burden (TMB) and are more likely to respond favorably to immune checkpoint inhibitors. However, further molecular testing is necessary to assess whether this is linked to Lynch syndrome (40). In this particular case, the patient’s tumor cells were negative for PMS2, while stromal cells remained positive. This pattern suggests a partial MMR deficiency, potentially linked to MLH1 mutations, given that PMS2 stability is dependent on its interaction with MLH1. To refine the treatment approach, additional molecular analysis using PCR or next-generation sequencing (NGS) is essential to confirm MSI status and identify relevant mutations (41).

The patient in this case has successfully undergone right hemicolectomy for colon cancer combined with pancreaticoduodenectomy. However, for future cases of synchronous right hemicolectal cancer and MSI-H duodenal cancer, it is crucial to explore novel treatment pathways based on immunotherapy to optimize treatment strategies, reduce surgical trauma and complications, and ultimately improve the patient’s quality of life and prognosis. Studies have indicated that tumors with microsatellite instability-high (MSI-H) are particularly responsive to immune checkpoint inhibitors, such as PD-1 inhibitors.

Studies have shown that MSI-H tumors are highly sensitive to immune checkpoint inhibitors (such as PD-1 inhibitors), and neoadjuvant immunotherapy can significantly reduce tumor burden, even achieving pathological complete remission (14). Based on this, a two-stage treatment plan can be proposed for similar future cases. The initial step should prioritize right hemicolectomy to manage MSS tumors while obtaining pathological staging and molecular test results, which will help determine the appropriate adjuvant treatment plan. After surgery, immunotherapy for MSI-H duodenal cancer can be considered to address local lesions and possibly avoid the risks associated with pancreatoduodenectomy (10). If imaging and endoscopic evaluations show complete remission, a “watchful waiting” strategy can be adopted. However, if partial response with significant tumor shrinkage is observed, local resection can be considered to further reduce surgical trauma.

Another possible approach is to directly implement neoadjuvant immunotherapy ± chemotherapy after confirming the diagnosis, especially when the patient’s overall condition is not ideal or the surgical risk is high. Existing studies have shown that MSI-H/dMMR tumors can achieve higher pathological response rates and long-term survival benefits through this strategy (42). If imaging examinations after neoadjuvant treatment show significant tumor shrinkage and confinement to resectable areas, further local surgery can be performed; if the lesions have completely disappeared, immunotherapy can be continued with close follow-up to reduce treatment burden and potential complications.

In summary, for the treatment of such complex cases of double primary cancer in the future, an interdisciplinary team (MDT) approach is crucial. It is necessary to integrate the patient’s molecular characteristics, organ function reserve, and expected survival benefits to develop an accurate personalized treatment plan. By optimizing the use of immunotherapy and the timing of surgery, we can reduce surgical trauma while improving overall efficacy and quality of life. Future research should focus more on the application of immunotherapy in such cases, providing further evidence-based guidance for clinical practice.

## Conclusion

Primary colorectal cancer combined with small bowel cancer, as double primary malignancies, presents a rare and challenging clinical scenario. The significant differences in their underlying mechanisms and molecular characteristics complicate both diagnosis and treatment. For early-stage cases, surgery remains the primary treatment option. In advanced stages, the strong response of MSI-H/dMMR subtypes to immunotherapy offers a promising path for precision medicine.

When simultaneous surgery is possible, it can considerably shorten the overall treatment timeline. However, for high-risk patients, staged surgery is a safer choice. The potential of neoadjuvant immunotherapy in MSI-H/dMMR tumors presents new possibilities for refining surgical strategies and improving long-term outcomes.

Effectively managing double primary cancers requires a coordinated, multidisciplinary approach. By integrating molecular diagnostics, advanced imaging techniques, and patient-centered care, healthcare providers can develop personalized and effective treatment plans. Future research should continue to explore how precision medicine can address the challenges posed by mismatch repair heterogeneity, ultimately improving patient survival and quality of life.

## Data Availability

The raw data supporting the conclusions of this article will be made available by the authors, without undue reservation.
